# Common primary fibroblastic growth factor receptor-related craniosynostosis syndromes: A pictorial review

**DOI:** 10.4103/1817-1745.66685

**Published:** 2010

**Authors:** Rohit K. Singh, Jitendra Singh Verma, Arun K. Srivastava, Awadhesh K. Jaiswal, Sanjay Behari

**Affiliations:** Department of Neurosurgery, Sanjay Gandhi Postgraduate Institute of Medical Sciences, Lucknow, India

**Keywords:** Apert syndrome, craniofacial, craniosynostosis, Crouzon syndrome, fibroblastic growth factor receptor, Pfeiffer syndrome

## Abstract

Mutations in different types of fibroblastic growth factor receptors (FGFRs) have been associated with a variety of phenotype abnormalities, the common ones being Apert, Crouzon and Pfeiffer syndromes. In this study, we present two representative cases having the Apert and Pfeiffer syndromes, respectively, and discuss their clinical presentation, sequel and surgical implications.

## Introduction

Fibroblastic growth factors (FGF) are a conglomerate of 22 known signal molecules that help in the regulation of self-proliferation, differentiation and migration pathways. Their action occurs through the FGF receptors (FGFRs) that consist of four tyrosine kinase receptors binding to the FGFR nonspecifically. Mutations in different types of FGFR receptors has been associated with a variety of phenotype abnormalities, e.g., FGFR1 mutation has been associated with the relatively milder form of Pfeiffer syndrome type 1 and mutations in FGFR2 and FGFR3 have been associated with the Apert, Crouzon and Pfeiffer syndromes.[1]

In this study, we present two representative cases having the Apert and Pfeiffer syndromes respectively and discuss their clinical implications.

## Case Report

### Case 1: Apert Syndrome

**Figure 1:**
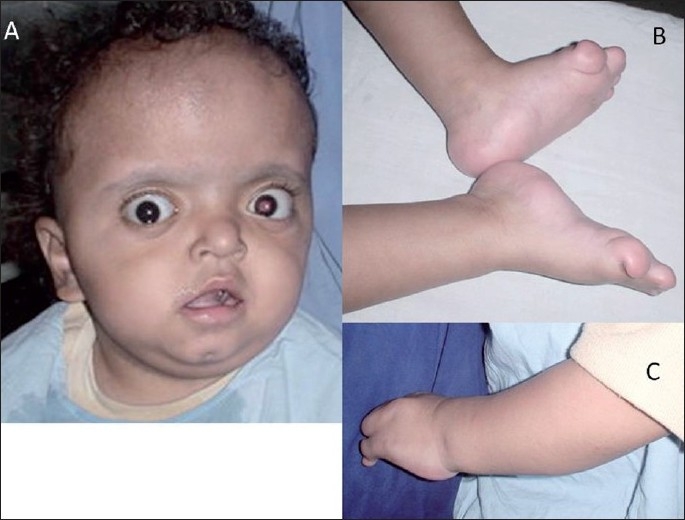
Case 1:(A) Crouzon’s syndrome with bilateral proptosis, fusion and elevation of the coronal suture, open anterior fontanelle and brachycephaly. **(B and C)** “Mitten-type” syndactyly of the fingers and toes with a broad and short thumb are seen

**Figure 2:**
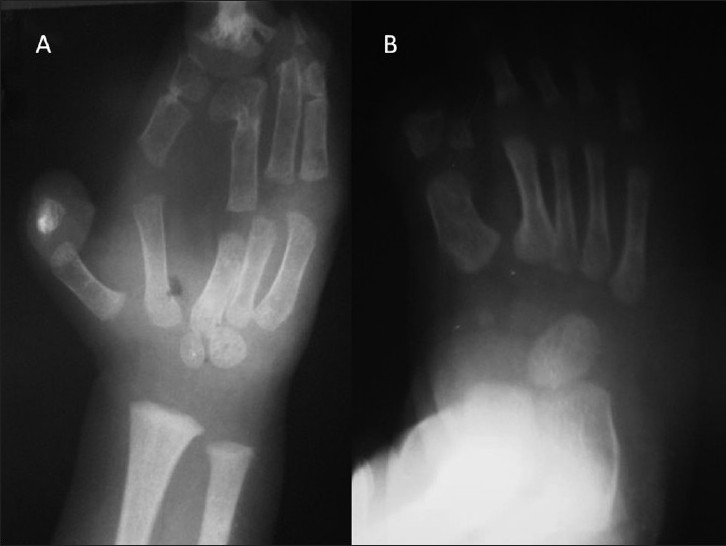
Case 1: (A) X-ray of the hand showing bony and soft tissue syndactyly with a broad, radially deviated thumb. **(B)** X-ray of the foot showing soft tissue syndactyly

**Figure 3:**
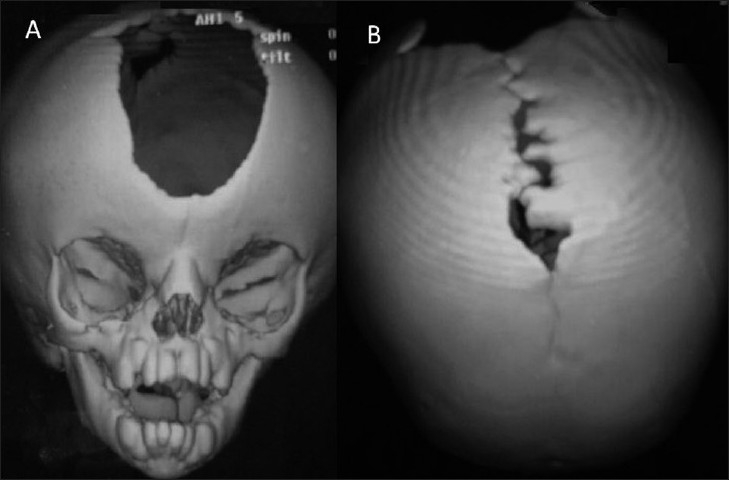
Case 1: (A and B) Three-dimensional reconstructed computed tomography scan showing a widely open anterior fontanelle with an open sagittal suture, bilateral coronal synostosis and a brachycephalic head

**Figure 4:**
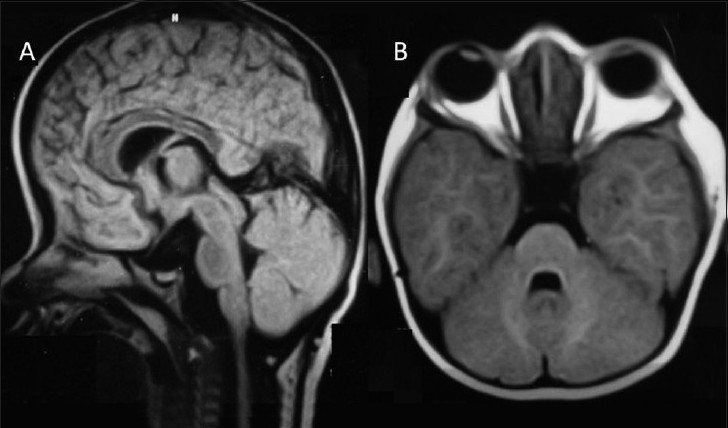
Case 1: (A and B) Magnetic resonance imaging revealed shallow orbits with bilateral proptosis, no Chiari malformation or hydrocephalus

This 4-month-old male child presented with bilateral proptosis, fusion and elevation of coronal suture, open anterior fontanelle and brachycephaly [Figure 1A]. He also had mitten-type syndactyly of his fingers and toes with a broad and short thumb [Figure 1B and C]. An X-ray of his hand showed bony and soft tissue syndactyly of the index, middle and ring fingers with a broad, radially deviated thumb [Figure 2A]. The X-ray of the leg showed soft tissue syndactyly and a short, medially deviated great toe [Figure 2B]. The computed tomographic (CT) three-dimensional reconstruction of the skull showed a widely open anterior fontanelle with an open sagittal suture, bilateral coronal synostosis and a brachycephalic head [Figure 3A and B]. Magnetic resonance imaging (MRI) of the head revealed shallow orbits with bilateral proptosis, no Chiari malformation or hydrocephalus [Figure 4A and B]. The patient’s parent did not opt for a prophylactic or cosmetic craniofacial surgery. They were, therefore, advised to come for regular follow-up.

### Case 2: Pfeiffer Syndrome Type II with Hydrocephalus

**Figure 5:**
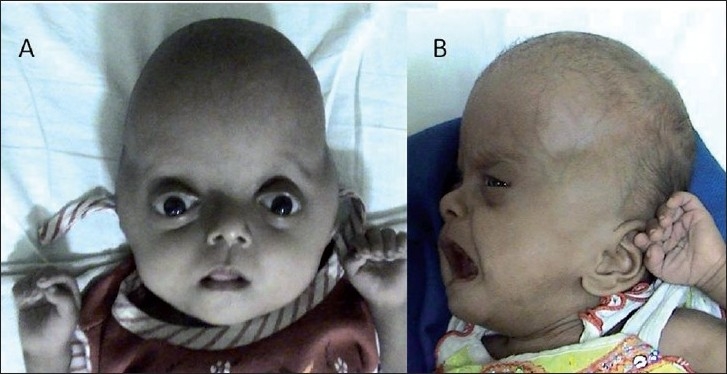
Case 2: (A and B) Pfeiffer’s syndrome type II with a clover leaf head, with bulging anterior fontanelle and bilateral temporal bulge, bilateral proptosis midfacial hypoplasia and broad and short thumb

**Figure 6:**
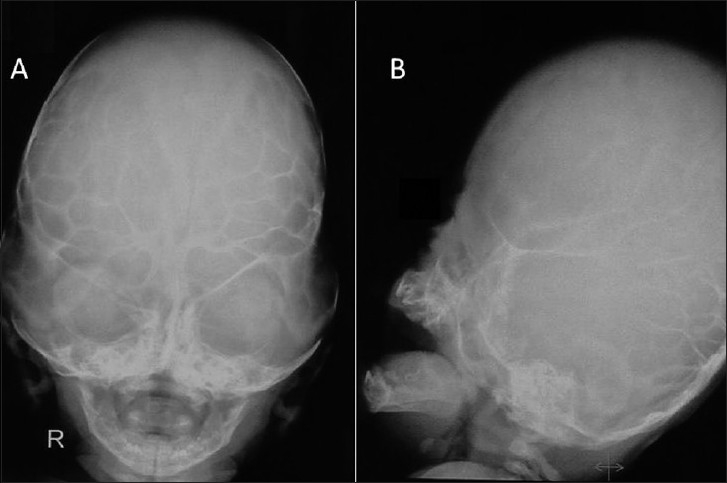
Case 2: (A and B) X-ray (anteroposterior and lateral images) of the skull showed a marked brachycephalic head with midfacial hypoplasia, silver-beaten appearance of the vault and elevation of sphenoid wings.

**Figure 7:**
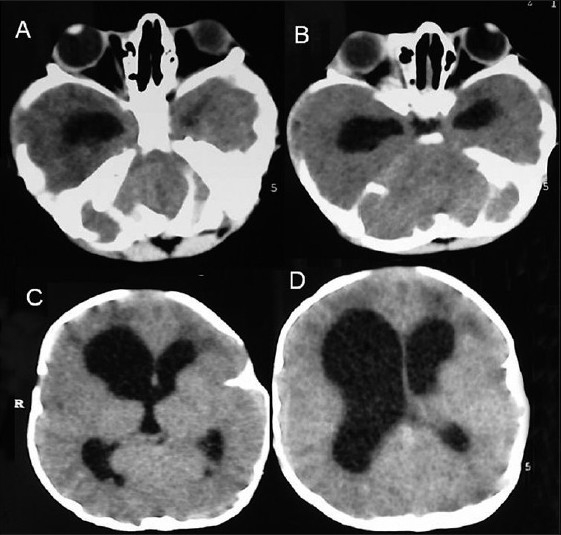
Case 2: (A–D) Computed tomography scan showing an asymmetrical ventricular dilatation (right lateral ventricle more than the left one). The fourth ventricle was not visualized. There is bilateral proptosis with shallow orbits

**Figure 8:**
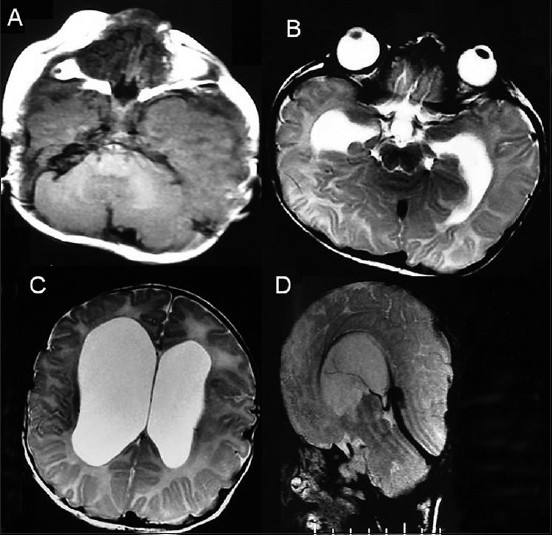
Case 2: (A–D) Magnetic resonance images showing gross ventriculomegaly with periventricular lucency and a chinked fourth ventricle. The sagittal image showed a steep angulation of the tent and straight sinus and a brachycephalic head

This 3-month-old female child presented with a clover leaf head with a bulging anterior fontanelle, bilateral temporal bulge, tense and prominent scalp veins, bilateral proptosis with adequate eye closure, midfacial hypoplasia and broad and short great toe and thumb [Figure 5A and B]. An X-ray (anteroposterior and lateral images) of her skull showed a marked brachycephalic head with midfacial hypoplasia, silver-beaten appearance of the vault and elevation of sphenoid wings indicative of basal craniosynostosis [Figure 6A and B]. Her CT revealed an asymmetrical ventricular dilatation (right lateral ventricle more than the left one). The fourth ventricle was not visualized. There was bilateral proptosis with shallow orbits [Figure 7A–D]. MRI images showed gross ventriculomegaly with periventricular lucency and a chinked fourth ventricle. The sagittal image showed a steep angulation of the tent and straight sinus and a brachycephalic head [Figure 8A–D]. In view of the gross hydrocephalus, the patient underwent a “Chabbra” right ventriculoperitoneal shunt. Her postoperative course was uneventful. At follow-up after 1.5 months, the anterior fontanelle was lax and the shunt was functioning digitally.

## Discussion

The characteristic skull shape in FGFR-related craniosynostosis syndromes is related to the growth of the skull in planes parallel to the prematurely closed sutures but not perpendicular to them. Coronal synostosis causes the skull to be brachycephalic (seen in our first patient) or turribrachycephalic (tower skull). A cloverleaf skull (kleeblatschadel) is due to coronal, lambdoidal, metopic and sagittal sutural synostosis, causing a trilobar skull deformity with the brain protruding through the open anterior and parietal fontanels (observed in our second patient). Lambdoidal or sagittal synostosis excludes a diagnosis of FGFR-related craniosynostosis even in the presence of hand and foot abnormalities. A metopic synostosis that results in trigonocephaly may occur as an isolated abnormality; its presence with other structure involvement, however, warrants exclusion of FGFR-related syndromes. The facial features common to all the syndromes include ocular hypertelorism, proptosis, midface hypoplasia, small beaked nose and mandibular prognathism.[1]

The specific clinical features in our first patient corresponded to the Apert syndrome. This syndrome has the highest risk of developmental delay and mental retardation (in almost 50% of patients). Turribrachycephalic skull with moderate to severe midface hypoplasia and soft tissue and bony (mitten glove) syndactyly of the fingers and toes and fused cervical vertebrae are the common findings. Hydrocephalus may occasionally be present.

Pfeiffer syndrome has been divided into three types by Cohen in 1993, with types 2 and 3 being more common and severe than type 1.[2] Pfeiffer syndrome type 1 is characterized by normal intellect, moderate to severe midface hypoplasia, medially deviated thumb and great toe, brachydactyly and occasional hydrocephalus and hearing loss. Pfeiffer syndrome type 2 is characterized by occasional developmental delay and mental retardation, characteristic clover leaf skull with proptosis (the patient may often be unable to close the eyes) and broad and medially deviated thumb and great toe with brachydactyly. Pfeiffer syndrome type 3 has the most severe manifestations, with clinical features of type 2, except that the skull is turribrachycephalic with coexisting extreme proptosis. In both Pfeiffer types 2 and 3, there may be choanal stenosis/atresia, laryngotracheal abnormalities, hydrocephalus, seizures, sacrococcygeal eversion and increased risk of early death.[1] Our second patient therefore corresponded to Pfeiffer type 2 in view of his clover leaf skull. None of the other life-threatening clinical symptoms, however, were present.

The special clinical feature of Crouzon syndrome include a normal intellect and craniofacial abnormalities like significant proptosis, external strabismus and mandible prognathism, but with normal extremities. The risk of intracranial hypertension is the highest in Crouzon’s syndrome, and progressive hydrocephalus may occur.[1] All these syndromes have an autosomal-dominant inheritance with complete penetrance.

Both our patients had a normal intellect. Although both of them had marked orbital stenosis resulting in proptosis and midfacial protrusion, their eyelid closure was adequate. The foreshortened orbit may result in an antimongloid obliquity of the palpebral fissure, lid retraction, global prolapse and corneal scarring, with the risk of visual impairment. Tarsorraphy, canthal ligament release and levator lengthening may be the short-term procedures required for orbital proptosis. Progressive proptosis may ultimately warrant an orbital advancement and expansion. Facial hypoplasia (and often associated choanal atresia or stenosis) may result in malocclusion, upper airway obstruction and obstructive sleep apnea. Airway obstruction may occur at a higher level due to midfacial retrusion, or at a lower level due to cartilage sleeve abnormality affecting the trachea–bronchial tree.[1] Chronic hypoxemia with resultant increased intracranial pressure may lead to impaired neural and intellectual development. Apart from a tracheostomy, surgical enlargement of the nasopharyngeal space, resection of the soft palate posterior to the levator–palati muscle, with or without an adenotonsillectomy, may also help in these circumstances.[3,4] In a persistent upper airway obstruction, midfacial advancement may be used. Tracheal stenosis, usually diagnosed by a bronchoscopy, may require a tracheal sleeve resection. Nocturnal monitoring of oxygen saturation is essential for these syndromes. Bilateral conduction type of hearing loss may occur due to ankylosis of the auditory ossicular chain. Hearing deficit may also occur due to mid ear effusion due to a stenotic nasopharyngeal space.[5] Vault expansion may be needed for the severely affected subtypes having craniostenosis, and is usually confined to the midvault, especially in patients having a cloverleaf deformity and coronal compression with severe skull constriction. Typical in Pfeiffer syndrome, the skull may assume a “Swiss cheese” lattice pattern due to raised intracranial pressure as a consequence of multiple sutural synostoses. The keel of the bone arising from this lattice work may extend deep into the brain, thinning the duramater and increasing the propensity for dural tear during the extradural surgery for vault expansion. The dural thinning with ventriculomegaly may increase the chances of cerebrospinal fluid leak during surgery for vault expansion. Venous hypertension may occasionally occur from the prominent scalp and bridging veins. During craniofacial advancement, excessive bleeding or fatal brain edema may result following coagulation or ligation of the emissary veins.[6]

Vertebral anomalies and Chiari malformation are also commonly associated with these syndromes. The latter may be congenital or acquired. The usual causes of death in these patients include sleep apnea, upper respiratory tract infection, undiagnosed, uncontrolled hydrocephalus with secondary Chiari malformation[7] and tracheal stenosis and midfacial hypoplasia leading to hypoxemia.

## Conclusion

A summary of the commonly encountered bicoronal primary FGFR-related craniosynostosis syndromes and their differentiating features and sequel are presented. A cerebrospinal fluid diversion procedure to control the raised intracranial pressure, tarsorraphy and orbital expansion procedures to prevent visual loss, an aggressive airway management and treatment of Chiari malformation by posterior decompression significantly delays the incidence of early mortality in these patients.
